# End - users’ perception of quality of care of children attending children’s outpatients clinics of University of Nigeria Teaching Hospital Ituku - Ozalla Enugu

**DOI:** 10.1186/1756-0500-7-800

**Published:** 2014-11-15

**Authors:** Christopher Bismarck Eke, Roland Chidi Ibekwe, Vivian Uzoamaka Muoneke, Josephat Maduabuchi Chinawa, MaryAnn Ugochi Ibekwe, Oluchi Mildred Ukoha, Bede Chidozie Ibe

**Affiliations:** Department of Paediatrics, College of Medicine University of Nigeria Enugu Campus, Nigeria/University of Nigeria Teaching Hospital Ituku - Ozalla, Enugu, Nigeria; Department of Paediatrics Federal Teaching Hospital Abakaliki, Abakaliki, Ebonyi State Nigeria; Department of Paediatrics University of Nigeria Teaching Hospital Ituku- Ozalla, Enugu, Nigeria

**Keywords:** End-users, Children, Perception, Quality of care

## Abstract

**Background:**

Knowledge of the specific details of end-users actual experiences with health system helps to identify areas for improvement in ways that standardized satisfaction measures are less able to provide in order to save lives, uphold public confidence and trust in healthcare delivery.

The aim of the study was to assess the end-users’ perception of the quality of clinical services rendered to children attending paediatric out-patient clinics of University of Nigeria Teaching Hospital, Ituku – Ozalla, Enugu.

**Methods:**

A cross sectional descriptive study was undertaken using exit point interviewer administered pre-tested/semi-structured questionnaire.

Assessment of perception of quality of care was undertaken in three service areas; waiting time, attitude of staff and comfort of the waiting hall. Data was analyzed using SPSS 16.0 and presented as percentages. Chi-square was used to compare means (p < 0.05).

**Results:**

A total of 367 respondents were interviewed. Over 50% of them were generally satisfied with overall quality of care. 329 (89.6%) were very satisfied with quality of doctors’ services, while the least satisfaction was with the quality of medical records services 139 (37.9%). Majority of the respondents 197 (53.7%) spent between 3–6 hours for each clinic visit and most of the waiting time spent was in the medical records and consultation.

**Conclusion:**

The care – givers perception of the general quality of care was adjudged high. However, overall waiting time was perceived to be unsatisfactory.

Efforts should be made to reduce the time spent by clients while accessing care in the facility.

**Electronic supplementary material:**

The online version of this article (doi:10.1186/1756-0500-7-800) contains supplementary material, which is available to authorized users.

## Background

Quality of health care is the degree to which health services for individuals and populations increase the likelihood of desired health outcomes [1]. And so the assessment of the quality of health-care therefore has become increasingly important to providers, regulators, and purchases of healthcare.

Recently healthcare providers are more interested in evidence – based medicine while purchasers of healthcare have shifted their focus to the cost effectiveness of health service delivery as well as the likely health outcomes [2].

And for paediatric health-care which involves the unique physician-parent-patient relationship with medical decisions made with the rights and obligations of each of these individuals kept in mind as well as an understanding of the ethical principles [3], assessment of satisfaction with the quality of care appears more complex.

These factors contribute to the limitation encountered when developing measures to assess the quality of care for children. The movement of a child through the various stages of development makes it difficult to establish what constitutes a “normal” outcome and by extension what constitutes a poor outcome.

Additional challenge encountered when measuring quality of care for children is that, in most cases, children depend on adults to both obtain care and to report on the outcomes of that care [4].

In some other circumstances, parents and their children may have different perceptions of what defines health or have different levels of satisfaction with the care they receive.

Children particularly those with special needs also depend on a broad range of services including medical system, community intervention, social as well as school based programmes.

And so, dependency on these various services increases the difficulty of measuring and appropriately attributing health outcomes observed in children to a particular service delivery entity [5].

Adolescents particularly depend on adults especially parents for access to some of their care; with regards to their special needs related to confidentiality and parent–child information sharing [5].

In general, patients’ perception of quality of care affect also their health behavior even after discharge and positive rating of service quality seems to be correlated with no hesitation about re-visiting or recommending the same hospital to someone else [1].

There has been an increase in medical tourism by Nigerians to India and other emerging countries for better and quality health services with huge economic costs and other associated sufferings to those involved.

Assessment of satisfaction with quality of care among end – users therefore is important for public policy analysts, healthcare managers, practitioners and users as well particularly to restore public confidence in our practice. This satisfaction can be measured indirectly by asking users to rate the quality of services they have received, or report their experience [6].

The results could be applied to help to improve the quality of health care delivery and uphold public confidence.

And so the objective of the current study was to assess end users’ perception of quality of care received by children attending paediatric outpatient clinics, in University of Nigeria Teaching Hospital (UNTH) Ituku - Ozalla, Enugu.

## Methods

The study was conducted at the children’s outpatient clinic (CHOP) of University of Nigeria Teaching Hospital (UNTH), Ituku- Ozalla, Enugu South East Nigeria between 1st February to 30th June, 2013. UNTH, Enugu is a five hundred bedded reference tertiary health facility in Enugu State, South East Nigeria. It serves over the three million citizens of Enugu State [7] and is a referral centre to the neighbouring south eastern states of Nigeria. The CHOP renders primary, secondary as well as tertiary healthcare services to children obtainable in the general out patients’ clinic, consultant/specialist clinics every weekday and it is the initial port of call of new patients except emergencies which usually go to the emergency unit or the new born special care unit as the case may be.

UNTH, Enugu is a national cardiothoracic centre of excellence and the Department of Paediatrics has won awards as the most organized department in the institution. Currently the department has about twenty eight consultants including 6 professors, 28 senior registrars, 22 registrars and about 30–40 house-officers who are constantly on three monthly rotations. There are 8 nurses with one of them having a certified training in paediatric nursing working in the children’s outpatients (CHOP) clinics in addition to 2 pharmacists and one pharmacy technician, 4 medical records officers and orderlies. There is a pharmacy and medical records unit attached to the CHOP in addition to dietician, public health nurse visitor/counselor.

Basically children attending the CHOP receive medical services from six service points namely – the medical records (where case record files are opened for the first timers or retrieved for re-visit clients), the nurses’ bay, the doctors’ consulting offices, the pharmacy and the laboratory. The waiting hall in the clinic area has a play station furnished with beautiful toys, indoor play items, television. It also has wooden benches with back rests where the parents and children who came for the clinic sit in addition to the nurses’ bay located at the centre of a long open space separating the consulting rooms.

Ethical clearance for the study was obtained from the Health Research Ethics Committee of UNTH, Enugu.

### Study design

A cross-sectional descriptive study was undertaken. About 60 children and their parents/caregiver respectively attend the outpatient clinic (CHOP) on each clinic day.

The respondents (parents/caregivers) were selected consecutively upon their informed consent using a non- ambiguous interviewer administered, pretested and semi- structured questionnaire. About 372 respondents were recruited out of which five voluntarily dropped off before the completion of their respective questionnaires giving an overall response rate of 98.7%. The questionnaire used was a modification of the tool applied by Ogbonnaya and Ogbonnaya [8] in Abakaliki, Ebonyi State Southeast Nigeria in a similar study. An outline of the questionnaire is added as in Additional file 1.

Social class was determined using the method proposed by Oyedeji [9] in Ilesha, South West Nigeria.

Data was analyzed using SPSS version 16.0 and presented in form of percentages and tables. Test of association was done using chi-square while the level of significance was set at p < 0.05.

## Results

A total of three hundred and sixty seven respondents were interviewed. The age distribution of the respondents ranged from 19 years to 67 years with majority of them 179 (48.9%) being in the 31–40 years age bracket. 284 (77.4%) of the respondents were females while 83 (22.6%) were males giving a male to female ratio of 1:3.4. Similarly a greater proportion of the interviewees were of Christian religious backgrounds 98.1% (360) while 309 (84.2%) were married. Majority of the respondents were in the upper social class (classes I and II) representing 63.8% of the total. The above socio-demographic characteristics are as shown in Table 1.

**Table 1:** **Socio-demographic characteristics of respondents**

Variables	Frequency (n = 367)	Percent (%)
**Age (years):**		
≤ 20	11	3-0
21- 30	108	29.4
31 – 40	179	48.8
41 – 50	60	16.4
51 – 60	6	1.6
61 – 70	3	0.8
**Gender:**		
Female	284	77.4
Male	83	22.6
Religion:		
ATR	3	0.8
Christianity	360	98.1
Islam	4	1.1
**Marital status:**		
Married	309	84.2
Widow/widower	31	8.4
Separated	6	1.6
Single	21	5.7
**SEC:**		
1	99	27.0
2	135	36.8
3	72	19.6
4	40	10.9
5	21	5.7

KEY: ATR – African Traditional Religion; SEC-Socioeconomic class.

Among the 367 respondents interviewed, majority were re-visit cases 243 (66.2%) while 124 (33.8%) were first time callers to the hospital. About one hundred and eighty eight of the respondents (51.2%) were satisfied with the overall quality of care (attitude of staff, comfort of waiting hall and the waiting time) in the CHOP while 329 (89.6%) were satisfied with the quality of doctors’ services. Concerning satisfaction with aspects of doctors’ service, 150 (40.9%) strongly agreed that they were directly involved in the making of management decisions on their children’s medical care while less than half of the respondents 141 (38.4%) strongly felt that the doctors ensured maximum courtesy in the course of their dealing with them. This is as shown in Table 2.

**Table 2:** **Satisfaction with aspects of doctor’s services**

Variables	Frequency (n = 367)	Percent (%)
Direct involvement about decisions on child’s medical care:		
Disagree	72	19.6
Agree	145	39.5
Strongly agree	150	40.9
Courtesy ensured:		
Disagree	55	15.0
Agree	171	46.6
Strongly agree	141	38.4
Consultation not rushed:		
Yes	301	82.0
No	66	18.0
Privacy ensured:		
Yes	286	77.9
No	81	22.1
Examination not causing pain:		
Yes	276	75.2
No	91	24.8

This is in sharp contrast to the 301 (82.0) that reported that the attending doctors did not rush their consultation. Similarly 286 (77.9%) felt that doctors ensured adequate privacy. In a likewise manner about two-thirds of the caregivers interviewed, 276 (75.2%) were of the opinion that the physical examination conducted on their wards by the respective attending physicians did not cause them pain (See Table 2).

On satisfaction with other healthcare staff, the respondents were least satisfied with the quality of the medical records 139 (37.9%), followed by nursing services 149(40.6%), laboratory services 153 (41.7%) and pharmacy 155 (42.2%) respectively as shown in Table 3. Only a small fraction of the respondents 67 (18.3%) felt that the waiting hall was very comfortable. It was observed that majority of the respondents 197 (53.7%) spent about 3–6 hours overall for each clinic visit while about 28 (7.6%) admitted spending more than 6 hours per visit as shown in Table 4, with most of the time spent on waiting for consultation (47.9%) 176 and retrieval of folders from the medical records.

**Table 3:** **Satisfaction with attitude of other health staff and comfort of the waitinghall**

Variables	Frequency (n = 367)	Percent (%)
**Medical records:**		
Satisfied	139	37.9
Somehow satisfied	163	44.4
Not satisfied	65	17.7
**Nursing services:**		
Satisfied	149	40.6
Somehow satisfied	167	45.5
Not satisfied	51	13.9
**Laboratory service**		
Satisfied	153	41.7
Somehow satisfied	132	36.0
Not satisfied	82	22.3
**Pharmacy:**		
Satisfied	155	42.2
Somehow satisfied	174	47.4
Not satisfied	38	10.4
**Comfort of the waiting hall:**		
Very comfortable	67	18.3
Somehow Comfortable	237	64.6
Uncomfortable	63	17.2

**Table 4:** **Satisfaction with waiting time**

Variables	Frequency (n = 367)	Percent (%)
Overall waiting time:		
< 3 hours	142	38.7
3-6 hours	197	53.7
> 6 hours	28	7.6
Medical records:		
Less than 30 minutes	63	17.2
30 – 60 minutes	128	34.9
Greater than 60 minutes	176	47.9
Waiting for consultation:		
Less than 30 minutes	91	24.8
30 – 60 minutes	169	46.1
Greater than 60 minutes	107	29.8
Pharmacy waiting time:		
Less than 30 minutes	151	41.1
30 – 60 minutes	112	30.5
Greater than 60 minutes	104	28.3

More than half of the respondents 241 (65.7%) were happy with the quality of care with only 13.4% (49) requesting for a change in the quality of care. On comparison of perception of quality of care among first time visitors and re-visit parents, there was no statistical significant difference (x^2^ = p = 2.502; 0.43).

Majority of the interviewees 202 (55.0%) recommended change(s) with duration of the overall waiting time in the hospital visit. This is followed by the duration of time spent in the medical records 199 (54.2%) as shown in Table 5.

**Table 5:** **Aspects of care that respondents recommended change(s)**

	Variables	Frequency (N = 367)	Percent (%)
A.	Waiting time:		
	Yes	202	55.0
	No	165	45.0
B.	Quality of medical treatment:		
	Yes	126	34.3
	No	241	65.7
C.	Medical records:		
	Yes	199	54.2
	No	168	45.8
D.	Nursing services:		
	Yes	151	43.8
	No	216	56.1
E.	Laboratory services:		
	Yes	146	39.8
	No	221	60.2
F.	Pharmaceutical services:		
	Yes	151	40.1
	No	216	58.9
G.	Doctors services:		
	Yes	95	25.9
	No	272	74.1

## Discussion and conclusions

Respondents in the current study were generally satisfied with the overall quality of care (attitude of the health staff, waiting time while accessing care, and the comfort of the waiting hall) received by their children in the clinics as was reported by both first time callers and re-visit users of the hospital. Similar findings have been reported by other researchers [8, 10].

This could partly be due to their satisfaction with the quality of care leading to higher rate of retention and loyalty; and no hesitation about re-visiting the same hospital [11].

Most caregivers were satisfied with the different aspects of doctors’ services; viz direct involvement about decision making on their child’s medi-care, consultation not rushed, privacy maintained and physical (medical) examination on the child not causing pain in the index study. The very high level of satisfaction with the quality of doctors’ services as observed in the current study could be the reason for further patronage of the facility (more re-visits cases compared to first – time callers).

Traditionally, the quality of medical care has been described as its ability to increase the probability of desired patient outcomes and decreases the probability of undesired outcomes [12].

Most of the caregivers could have generally rated their care quality by the extent to which patients physiological functions have improved as a consequence of receiving medical care services.

However, more than half of the respondents felt that the doctors did not ensure adequate courtesy during their consultations. Greetings as well as adequate explanations concerning patients medical conditions including treatment options and possible outcomes could shape the perception of the quality of care by both patients and their caregivers. In addition courtesy gives one initial feeling of acceptance and makes them to be in more relaxed manner to report their medical histories.

Past studies indicate that patients cannot properly evaluate the outcome of healthcare services and the technical competence of practitioners, since they often lack sufficient expertise and skill to make such judgments [13]. Consequently, end- users’ might infer the level of technical quality based on non-technical aspects, such as care providers’ compassion and empathy, responsiveness and co-ordination of care among individual healthcare personnel [14].

Highly competent and professional healthcare personnel are required for high quality care and satisfied patients. Healthcare personnel must do their utmost to provide patients with person-centred care.

Patients desire respect, less stigma and providers taking time to listen to their concerns. Patients want to be involved in their care decisions. Similar findings were observed in the current study.

Our study indicated that ancillary service quality dimensions such as non-physician care and quality of the environment (waiting hall) were important for satisfaction formation of outpatients during their visit to the hospital. Majority of the respondents were generally satisfied with the present status of the ancillary services.

The interaction between the patients and other healthcare personnel including the nurses for instance is a determinant factor of their satisfaction. Interpersonal relationship includes honest, trust, respect, understanding, empathy, knowing individual as a person, touch, friendliness and feeling connected [15] and also knowledge of the patient in person.

A comfortable environment has had a positive impact on patients’ satisfaction ratings. Physical comfort, emotional support and respect for patient preferences were stronger predictors of patient’s overall evaluation of quality of care [16].

Less than a quarter of the caregivers in the index study perceived the waiting hall to be either very comfortable or somehow comfortable.

Waiting time is a vital segment of service quality. More than half of the respondents (53.7%) perceived the overall waiting time as being too long and unnecessary.

In the current study about 7.6% of the respondents reported that they spent over 6 hours during each hospital visit.

It follows then that the waiting time was the service quality dimension that most caregivers perceived as being unsatisfactory and needed improvements. Similar reports have been documented by other researchers [17, 18]. The service units with the longest duration of waiting time were in the consultation and medical records.

Waiting time has an impact on patient satisfaction. In a study conducted by West-away et al. [19], in South Africa, it is reported that in respect of a country setting/developed or not developed, the highest levels of dissatisfaction was with waiting time. Patients do not like to be left alone for a longtime [20].

A lot of time is usually wasted while waiting to retrieve old folders for revisit cases and sometimes some of the case record files are misplaced, causing a lot of delay.

Also, every Wednesday afternoon and Friday morning the department runs a clinical or mortality conference respectively and the patients have to wait till the end of the conference before clinics will resume. Hence prolonging the overall time spent by patients and their caregivers while accessing healthcare.

Finally, respondents recommended changes in the duration of the overall waiting time majorly.

In conclusion, the care – givers perception of the general quality of care was adjudged high. However, overall waiting time was perceived to be unsatisfactory.

Efforts should be made to reduce the time spent by clients while accessing care in the facility.

### Limitations

1. The out-patients sample population used in this study (compared to their in-patients counterparts who spend more time in the hospital), who leave the hospital environment as with the physician and non-physician care providers.
2. The study considered only the experiences of the parents and not those of the children. However, there is evidence that the parents’ perception of healthcare services is correlated to their children’s perception only to a limited degree.

### Research ethics

Ethical clearance for the study was obtained from the Health Research Ethics Committee of UNTH, Enugu while informed consents were obtained from the respondents.

## Authors’ information

UOM: Senior registrar I

ECB, MVU, CJM: Lecturer I/Honorary Consultant

IRC, IMU: Senior Lecturer/Honorary Consultant

IBC: Professor/Honorary Consultant

## Electronic supplementary material

Additional file 1: **Study questionnaire.** (DOCX 18 KB)

## Abbreviations

CHOP: Children’s out- patient clinics
UNTH: University of Nigeria Teaching Hospital.
