# Supplementary material for: End - users’ perception of quality of care of children attending children’s outpatients clinics of University of Nigeria Teaching Hospital Ituku - Ozalla Enugu
Source: BMC Res Notes. 2014 Nov 15;7:800. doi: 10.1186/1756-0500-7-800 (PMC4247624; doi:10.1186/1756-0500-7-800)
Supplement: Supplementary file 1 — Additional file 1: Study questionnaire. (DOCX 18 KB) [file 13104_2014_3342_MOESM1_ESM.docx]

QUESTIONNAIRE

ENDUSERS’ PERCEPTION OF QUALITY OF CARE BY CAREGIVERS OF CHILDREN

ATTENDING CHILDREN’S OUTPATIENT CLINICS IN UNIVERSITY OF NIGERIA

TEACHING HOSPITAL (UNTH), ITUKU -OZALLA, ENUGU.

Serial No: ……………………………… Hospital No……………………

**Socio-demographic characteristics of child’s parent/care- Giver:**

1. Age (in years as at last birthday): ……………………………………………………………………
2. Place of residence: …………………………………………………………………………………………
3. Phone Number: ……………………………………………………………………………………………
4. Gender: (a) Male (b) Female
5. Religion: (a) African Traditional Religion (b) Orthodox (c) Pentecostal (d) Muslim
6. **Parental Occupation (Using Oyedeji’s Criteria);**

**Father Mother**

1. Senior public servants, professional, manager, large

Scale trader, businessman or contractor.

1. Intermediate grade public servant, senior school

Teacher, nurse or technician.

1. Junior school teacher, clerk, auxiliary nurse, driver, mechanics.
2. Petty trader, laborer, messenger.
3. Unemployed, full time housewife, student or subsistence farmer
4. **Parental Education Attainment (Using Oyedeji’s Criteria):**

**Father**  **Mother**

1. University graduate or equivalents
2. School certificate holder (GCE or SSCE) who also
3. School certificate or Grade 11 teachers certificate

Holder or equivalent.

1. Junior Secondary school Certificate, Modern three

and primary.

1. One who cannot read or write or illiterate.

1. Visit frequency: (a) First Time visit (b) Revisit
2. Have you received treatment from our facility in the last three month?

Yes____________________ No____________________________

1. If Yes, you received treatment as
2. In- patient_______________ (b) Out-patient_______________
3. How can you describe your health condition after treatment from the

hospital? (a)Excellent___ (b) Good____ (c) Fair____ (d) Worse______

1. How can you rate the service you received from the healthcare professionals?

(a)Very Satisfactory____ (b) Somehow Satisfactory______ (c) Not too Satisfactory____ (d) Not Satisfactory_______.

13. **Are you satisfied with Doctor- patient relationship in this clinic?**

(a) Yes (b) No

**For the two questions below answer with Strongly Agree, Agree, Disagree, or Strongly Disagree.**

14. You were involved directly in decisions about your medical care?...................

15. How can you rate the courtesy and respect showed by the healthcare professionals who attended to you?__________________________.

16. What about the healthcare service would you like to be changed? ___________________________________

17. How do the healthcare professionals relate with the patients? _________________________

18. Aspects of Doctor- patient interaction:

(a) Consultation was not rushed (i) Yes (ii) No

(b) Privacy was ensured: (i) Yes (ii) No

(c) Doctor’s examination did not cause pain: (i) Yes (ii) No

19**. Aspects of overall/specific waiting time:**

**(a) Overall waiting time:** (i) < 3 hours (ii) 3 - 6 hours.

**(b) Medical Records:** (i) < 30 minutes (ii) 30 - 60 minutes (iii) > 60 minutes

**(c) Waiting hall:** (i) < 30 minutes (ii) 30 - 60 minutes (iii) > 60 minutes

**(d) Pharmacy waiting time**: (i) < 30 minutes (ii) 30 - 60 minutes (iii) > 60

minutes.

(e) **Medical Laboratory Waiting Time:** (i) < 30 minutes (ii) 30 - 60 minutes (iii) > 60 minutes.

20. **Perception of comfort/ quality of the waiting hall**

(a) Very comfortable (b) Comfortable (c) Very uncomfortable

21**. Quality of Pharmaceutical service:**

(a) Availability of essential drugs: (i) YES (ii) NO

(b) Cost of drugs: (i) Expensive (ii) Moderate (iii) Cheap

22. Perception of quality of outpatient laboratory service (With respect to duration of release of lab result): (a) Very Satisfactory __________ (b) Somehow satisfactory _______ (c) Not too satisfactory______(d) Not satisfactory___________

23. Perception of satisfactory with the other Health staff

(a) Pharmacist: (i) Very satisfactory__________________

(ii) Somehow satisfactory _______________________

(iii) Not too satisfactory ________________________

(iv) Not satisfactory ___________________________

(b)Nurse: (i) Very Satisfactory ____________________

(ii) Somehow satisfactory ____________________

(iii) Not too satisfactory _____________________

(iv) Not satisfactory ________________________

(c) Medical Records Officer: (i) Very Satisfactory __________________________

(ii) Somehow satisfactory ______________________

(iii) Not too satisfactory ________________________

(iv) Not satisfactory __________________________

18. If you an alternative health care facility would you still patronize this health facility: (a) Yes (b) No.

19. If you want to recommend a change(s) in any of the aspects of the services which one would you suggest?

(A) Nursing services: (a) Yes (b) No

(B) Laboratory services: (a) Yes (b) No

(C) Pharmaceutical services: (a) Yes (b) No

(D) Doctors services: (a) Yes (b) No

(E) Waiting Time: (a) Yes (b) No

(F) Quality of medical treatment: (a) Yes (b) No.
